# Association of the Interaction Between Familial Hypercholesterolemia Variants and Adherence to a Healthy Lifestyle With Risk of Coronary Artery Disease

**DOI:** 10.1001/jamanetworkopen.2022.2687

**Published:** 2022-03-16

**Authors:** Akl C. Fahed, Minxian Wang, Aniruddh P. Patel, Ezimamaka Ajufo, Dimitri J. Maamari, Krishna G. Aragam, Deanna G. Brockman, Trish Vosburg, Patrick T. Ellinor, Kenney Ng, Amit V. Khera

**Affiliations:** 1 Center for Genomic Medicine, Department of Medicine, Massachusetts General Hospital, Boston; 2 Division of Cardiology, Department of Medicine, Massachusetts General Hospital, Boston; 3 Cardiovascular Disease Initiative, Broad Institute of MIT and Harvard, Cambridge, Massachusetts; 4 Department of Medicine, Harvard Medical School, Boston, Massachusetts; 5 Department of Medicine, UT Southwestern Medical Center, Dallas, Houston, Texas; 6 Center for Computational Health, IBM Research, Cambridge, Massachusetts; 7 Verve Therapeutics, Cambridge, Massachusetts

## Abstract

**Importance:**

Familial hypercholesterolemia variants impair clearance of cholesterol from the circulation and increase risk of coronary artery disease (CAD). The extent to which adherence to a healthy lifestyle is associated with a lower risk of CAD in carriers and noncarriers of variants warrants further study.

**Objective:**

To assess the association of the interaction between familial hypercholesterolemia variants and adherence to a healthy lifestyle with risk of CAD.

**Design, Setting, and Participants:**

This cross-sectional study used 2 independent data sets with gene sequencing and lifestyle data from the UK Biobank: a case-control study of 4896 cases and 5279 controls and a cohort study of 39 920 participants. Participants were recruited from 22 sites across the UK between March 21, 2006, and October 1, 2010. The case-control study included participants with CAD and controls at enrollment. The cohort study used a convenience sample of individuals with available gene sequencing data. Statistical analysis was performed from April 2, 2019, to January 20, 2022.

**Exposures:**

Pathogenic or likely pathogenic DNA variants classified by a clinical laboratory geneticist and adherence to a healthy lifestyle based on a 4-point scoring system (1 point for each of the following: healthy diet, regular exercise, not smoking, and absence of obesity).

**Main Outcomes and Measures:**

Coronary artery disease, defined as myocardial infarction in the case-control study, and myocardial infarction, ischemic heart disease, or coronary revascularization procedure in the cohort study.

**Results:**

The case-control study included 10 175 participants (6828 men [67.1%]; mean [SD] age, 58.6 [7.2] years), and the cohort study included 39 920 participants (18 802 men [47.1%]; mean [SD] age at the end of follow-up, 66.4 [8.0] years). A variant was identified in 35 of 4896 cases (0.7%) and 12 of 5279 controls (0.2%), corresponding to an odds ratio of 3.0 (95% CI, 1.6-5.9), and a variant was identified in 108 individuals (0.3%) in the cohort study, in which the hazard ratio for CAD was 3.8 (95% CI, 2.5-5.8). However, this risk appeared to vary according to lifestyle categories in both carriers and noncarriers of familial hypercholesterolemia variants, without a significant interaction between carrier status and lifestyle (odds ratio, 1.2 [95% CI, 0.6-2.5]; *P* = .62). Among carriers, a favorable lifestyle conferred 86% lower risk of CAD compared with an unfavorable lifestyle (hazard ratio, 0.14 [95% CI, 0.04-0.41]). The estimated risk of CAD by the age of 75 years varied according to lifestyle, ranging from 10.2% among noncarriers with a favorable lifestyle to 24.0% among noncarriers with an unfavorable lifestyle and ranging from 34.5% among carriers with a favorable lifestyle to 66.2% among carriers with an unfavorable lifestyle.

**Conclusions and Relevance:**

This study suggests that, among carriers and noncarriers of a familial hypercholesterolemia variant, significant gradients in risk of CAD are noted according to adherence to a healthy lifestyle pattern. Similar to the general population, individuals who carry familial hypercholesterolemia variants are likely to benefit from lifestyle interventions to reduce their risk of CAD.

## Key Points

**Question:**

Is adherence to a healthy lifestyle associated with lower risk of coronary artery disease in carriers and noncarriers of pathogenic DNA variants in familial hypercholesterolemia–related genes?

**Findings:**

In a case-control study of 10 175 participants and cohort study of 39 920 participants, there was a significant risk gradient of coronary artery disease according to variant carrier and lifestyle categories. Estimated risk by the age of 75 years among variant carriers ranged from 35% for those with a favorable lifestyle to 66% for those with an unfavorable lifestyle.

**Meaning:**

These findings suggest that a favorable lifestyle is associated with a lower risk of coronary artery disease in carriers and noncarriers of familial hypercholesterolemia variants.

## Teaser

This cross-sectional study uses 2 data sets from the UK Biobank to assess the association of the interaction between familial hypercholesterolemia variants and adherence to a healthy lifestyle with risk of coronary artery disease (CAD).

## Introduction

Familial hypercholesterolemia (FH) is a mendelian disorder—caused by pathogenic DNA variants in any of 3 related genes (*LDLR* [OMIM 606945], *APOB* [OMIM 107730], or *PCSK9* [OMIM 607786])—characterized by increased low-density lipoprotein (LDL) cholesterol concentrations and risk of coronary artery disease (CAD).^1,2^ Gene sequencing in large study populations have identified FH variants in 0.2% to 0.5% of the general population, with a prevalence among patients with early-onset CAD of up to 2%.^3,4,5,6,7,8^ Despite growing recognition of the potential utility of identifying FH variant carriers early in life to enable targeted prevention, the condition remains underdiagnosed and undertreated within current clinical practice.^2,9,10,11^

Although the risk of CAD among carriers of a FH variant is thought to be largely caused by increased LDL cholesterol concentrations, lifestyle factors may also play an important role. In the general population—of whom more than 99% do not carry a FH variant—individuals who adhere to a healthy lifestyle have markedly lower rates of developing CAD.^12,13,14^ This observational evidence has led to an emphasis on the promotion of a healthy lifestyle as a population health strategy to decrease the public health burden of CAD.^15,16,17^ Healthy lifestyle factors include a healthy diet, regular exercise, avoidance of smoking, and maintaining a normal weight. Among those with increased genetic risk—as defined based on family history or an increased polygenic risk score—rates of CAD decreased by up to 50% among those who adhered to a healthy lifestyle.^18^ Whether a similar gradient exists among carriers of an FH variant has not been fully explored, to our knowledge.

Improved understanding of the association of the interaction between FH variants and adherence to a healthy lifestyle with risk of CAD may have 2 important implications. First, a more nuanced estimation of the risk of CAD for carriers of FH variants may inform shared decision-making about prevention strategies—such as intensity of LDL cholesterol lowering or prophylactic use of aspirin. Second, especially for carriers of a FH variant who may feel a sense of predeterminism for developing CAD,^19^ demonstration of a risk gradient according to lifestyle might help motivate adoption of a healthy lifestyle.

Here, we analyzed gene sequencing and lifestyle data in a case-control study of 10 175 individuals and cohort study of 39 920 individuals to evaluate the extent to which adherence to a healthy lifestyle attenuates risk of CAD among carriers and noncarriers of a FH variant.

## Methods

### Study Populations

We analyzed 2 independent study populations derived from the UK Biobank, which enrolled more than 500 000 participants aged 40 to 69 years between March 21, 2006, and October 1, 2010.^20^ Analysis of the UK Biobank data was approved by the Mass General Brigham institutional review board. Written informed consent was obtained from all participants by the UK Biobank. This study followed the Strengthening the Reporting of Observational Studies in Epidemiology (STROBE) reporting guideline.^21^

First, we designed a CAD case-control study of 10 175 unrelated participants, including 4896 participants who received a diagnosis of CAD prior to study enrollment and 5279 controls as previously described.^22^ Cases were identified based on the occurrence of myocardial infarction as defined centrally, using data from self-report at enrollment, hospitalization records, or death registry records.^23^ Controls included participants free of any self-reported or documented history of CAD, including myocardial infarction, coronary revascularization, or ischemic heart disease. From an original 12 909 samples that underwent gene sequencing, we excluded 30 samples that failed quality control, 27 because of sample relatedness, and 2677 with incomplete lifestyle data (eTable 1 and eFigure 1 in the Supplement).^22^

Second, we studied an independent cohort study of 39 920 additional UK Biobank participants with gene sequencing and lifestyle data available as previously described.^5,22^ Coronary artery disease was defined based on self-report of myocardial infarction, hospitalization records confirming a diagnosis of myocardial infarction or ischemic heart disease, coronary revascularization procedures, or death registry data indicating ischemic heart disease or myocardial infarction as a cause of death. From an original 49 960 samples that underwent gene sequencing, we excluded 222 samples that failed quality control, 926 because of sample relatedness, and 8892 with incomplete lifestyle data (eTable 2 and eFigure 1 in the Supplement).^22^ Participants excluded for incomplete lifestyle data were more likely to have CAD than the remaining study participants (eTables 1 and 2 in the Supplement).

In both study populations, LDL cholesterol concentrations were measured in biospecimens provided at time of study enrollment. For those who reported taking lipid-lowering medications at time of study enrollment (both as a category of “cholesterol lowering” and specifying the medication by name), we estimated untreated LDL cholesterol concentrations by adjusting concentrations depending on the type of lipid-lowering medication as described previously (eTable 3 in the Supplement).^5^

### Ascertainment of Carriers of an FH Variant

Within both studies, carriers of a pathogenic or likely pathogenic FH variant were identified using gene sequencing data according to current clinical criteria as previously described.^4,5,22^ In brief, we performed whole-exome sequencing in the CAD case-control study at the Broad Institute of MIT and Harvard (Cambridge, Massachusetts). Whole-exome sequencing of participants of the cohort study was performed by the Regeneron Genetics Center (Tarrytown, New York). Identified genetic variants in any of 3 genes causal for FH—*LDLR*, *PCSK9*, and *APOB*—were classified by a board-certified laboratory geneticist at the Mass General Brigham Laboratory for Molecular Medicine (Boston, Massachusetts) according to American College of Medical Genetics and Genomics/Association of Molecular Pathology criteria.^24^ These criteria include assessment of the effect of the variant on the gene product, any previous reports of pathogenicity of the variant, functional studies supporting the damaging effect of the gene, and the prevalence of the variant in cases with the disease and controls in prior studies.^24^ Geneticists were blinded to any clinical information about the variant carrier at time of classification.

### Assessment of Adherence to a Healthy Lifestyle

We adapted a 4-point score for the evaluation of adherence to a healthy lifestyle based on the American Heart Association’s strategic goals for cardiovascular health based on data available from UK Biobank participants and as described previously.^16,18,25,26^ One point was given for each of 4 favorable lifestyle characteristics as follows. (1) One point was given for healthy diet pattern, consistent with recently defined characteristics, by having at least 3 of the following: total fruit, 3 pieces or more per day; total vegetables, 12 or more heaping tablespoons per day (3 servings); oily fish twice per week or more; processed meat once per week or less; and red meat twice per week or less.^12,17^ It was also required that the participant’s diet had not changed in the 5 years prior to enrollment. This requirement was particularly relevant for the case-control study because most cases (3092 of 4896 [63.2%]) had received a diagnosis of CAD in the 5 years prior to enrollment, and we wanted to minimize bias introduced by changes in diet after CAD diagnosis. (2) One point was given for regular exercise, as defined by meeting recommendations of the Physical Activity Guidelines for Americans of 15 or more metabolic equivalent of task–hours per week.^27^ Metabolic equivalent of task–hours were calculated based on self-reported weekly duration of moderate and vigorous activity (in hours) assessed using the International Physical Activity Questionnaire multiplied by 4 and 8, respectively, to reflect expected energy expended,^28,29^ (3) One point was given for not currently smoking, as assessed by self-report. (4) One point was given for absence of obesity, as assessed by measured body mass index of less than 30 (calculated as weight in kilograms divided by height in meters squared) at time of study enrollment.^30^ A favorable lifestyle was defined as having 3 or 4 of the healthy lifestyle characteristics, an intermediate lifestyle as having 2, and an unfavorable lifestyle as having none or only 1 of the characteristics. To focus on adherence to a healthy lifestyle, measures of clinical risk factors that were also considered part of ideal cardiovascular health, specifically total cholesterol, blood pressure, and fasting plasma glucose levels, were not included in the score, consistent with prior work from our group and others.^18,25,26^

### Statistical Analysis

Statistical analysis was performed from April 2, 2019, to January 20, 2022. The association of FH variants and healthy lifestyle with LDL cholesterol concentrations was evaluated using a linear regression model adjusted for sex, age, age squared, and genetic ancestry as quantified by the first 4 principal components. In the case-control study, participants were stratified into 3 groups according to their healthy lifestyle score: favorable, intermediate, or unfavorable, as performed in a previous study.^18^ For carriers and noncarriers in each lifestyle score group (coded as an indicator variable with 6 levels, 2 carrier statuses × 3 lifestyle groups), the odds ratio (OR) for disease was calculated in a logistic regression model with age, sex, and the first 4 genetic principal components of ancestry as covariates.^31^ A sensitivity analysis including an additional covariate in the model for socioeconomic deprivation, the Townsend score, showed that the association of FH variant carrier status with CAD was unchanged and that the association of lifestyle score with CAD was only minimally attenuated.^32^ A multiplicative interaction was evaluated from the logistic regression model. An additive interaction was evaluated by calculating the relative excess risk due to interaction using the epiR package.^33^ For the cohort study, we quantified the age-dependent probability of disease in carriers of an FH variant and in noncarriers of different lifestyle scores. We fit a Cox proportional hazards regression model with age as the time scale, defining the time to event as the age at which the diagnosis was first ascertained among cases and the age at the most recent follow-up among controls as performed previously.^5^ The model included carrier status, lifestyle score, sex, and the first 4 principal components of ancestry as covariates. To estimate the probability of disease, we used the FH carrier and lifestyle score effects from the model and standardized the remaining covariates at their mean. The probability of disease by time *t* was estimated by *F*(*t*) = 1 − *S*(*t*), where *S*(*t*) is the survivor function, estimated by the *survfit* function from the R *survival* package.

Statistical analyses were performed using R software, version 3.5 (R Project for Statistical Computing). Statistical significance was set at *P* < .05, and 2-sided *P* values were used.

## Results

The case-control study included 10 175 participants (6828 men [67.1%]; mean [SD] age, 58.6 [7.2] years). The cohort study included 39 920 participants (18 802 men [47.1%]; mean [SD] age at the end of follow-up, 66.4 [8.0] years).

### FH Carrier Status, LDL Cholesterol, and Risk of CAD

**Figure 1.**
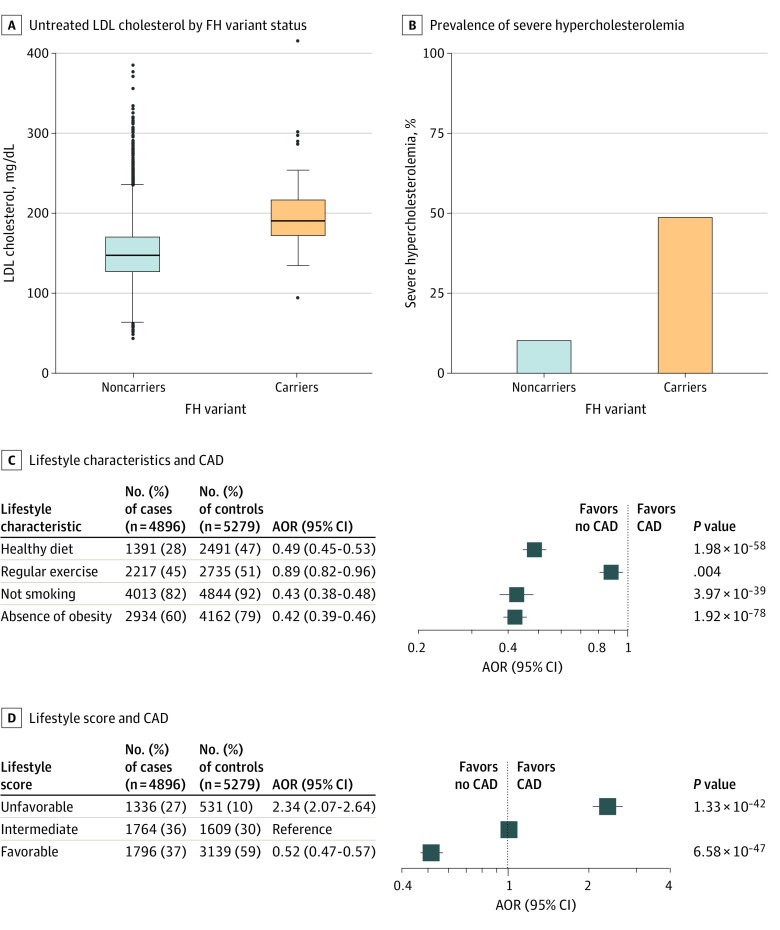
Data on Low-Density Lipoprotein (LDL) Cholesterol Levels and Healthy Lifestyle in the Case-Control Study (N = 10 175) A, Boxplot showing the distribution of estimated untreated LDL cholesterol levels according to familial hypercholesterolemia (FH) variant status. Low-density lipoprotein cholesterol levels were higher in FH variant carriers (n = 47) than in noncarriers (n = 10 128; *P* < .001). The ends of each box indicate the 25th and 75th percentiles; the horizontal line inside each box indicates the median, and the whiskers indicate the upper and lower adjacent values with the dots as outliers. B, Prevalence of severe hypercholesterolemia, defined as an estimated untreated LDL cholesterol level of 190 mg/dL or more by FH variant carrier status. Half of the variant carriers do not have severe hypercholesterolemia. The estimated untreated LDL cholesterol level was calculated by adjusting the measured LDL cholesterol level at enrollment for the effect size of lipid-lowering medications for individuals taking them (eTable 3 in the Supplement). C, Association of each healthy lifestyle characteristic with coronary artery disease (CAD). A sensitivity analysis considering both current and former smoking yielded comparable results (adjusted odds ratio [AOR], 0.44 [95% CI, 0.40-0.48]). D, Association of healthy lifestyle as measured by a 4-point scoring system (0-1 characteristic, unfavorable; 2 characteristics, intermediate; and 3-4 characteristics, favorable) with CAD. The AOR was calculated in a logistic regression model with age, sex, and genetic ancestry as defined by the first 4 genetic principal components as covariates.

Any of 24 pathogenic or likely pathogenic FH variants was identified in 35 of 4896 CAD cases (0.7%) and in 12 of 5279 controls (0.2%). These 24 variants included 21 variants in the *LDLR* gene, 1 in the *PCSK9* gene, and 2 in the *APOB* gene (eTable 4 in the Supplement). As expected, LDL cholesterol concentrations were significantly higher in carriers of an FH variant compared with noncarriers (untreated concentrations, 201 vs 148 mg/dL [to convert to millimoles per liter, multiply by 0.0259], corresponding to an adjusted difference of 53 mg/dL [95% CI, 48-58 mg/dL]; *P* < .001) (Figure 1A). A total of 22 of 45 carriers (48.9%) of an FH variant met clinical criteria for severe hypercholesterolemia (estimated untreated LDL cholesterol ≥190 mg/dL) compared with 951 of 9648 noncarriers (9.9%) (Figure 1B). Two carriers had missing data on LDL cholesterol levels. On average, those who carried a FH variant had 3-fold increased odds of CAD compared with noncarriers (adjusted OR, 3.0 [95% CI, 1.6-5.9]; *P* < .001). This association was unchanged even after adjusting for the lifestyle score (adjusted OR, 3.07 [95% CI, 1.56-6.06]; *P* = .001).

### Healthy Lifestyle Characteristics, LDL Cholesterol, and Risk of CAD

Each of the 4 healthy lifestyle characteristics was associated with a decreased risk of CAD among participants of the case-control study (adjusted ORs: healthy diet pattern, 0.49 [95% CI, 0.45-0.53]; regular exercise, 0.89 [95% CI, 0.82-0.96]; not smoking, 0.43 [95% CI, 0.38-0.48]; and absence of obesity, 0.42 [95% CI, 0.39-0.46]) (Table 1 and Figure 1C). Risk gradients were even more pronounced when the number of healthy lifestyle characteristics were considered in an aggregate lifestyle score (eFigure 2 in the Supplement). Compared with those with an intermediate lifestyle (score of 2), the OR for the risk of CAD for those with an unfavorable lifestyle (score of 0 or 1) was 2.34 (95% CI, 2.07-2.64), and the OR for the risk of CAD for those with a favorable lifestyle (score of 3 or 4) was 0.52 (95% CI, 0.47-0.57) (Figure 1D). Healthy lifestyle score was only minimally associated with LDL cholesterol concentrations. Compared with participants with an intermediate lifestyle, participants with a favorable lifestyle had 1.8 mg/dL (95% CI, 1.0-2.5 mg/dL) lower LDL cholesterol, and participants with an unfavorable lifestyle had 3.5 mg/dL (95% CI, 2.5-4.5 mg/dL) higher LDL cholesterol.

**Table 1.** Characteristics of Coronary Artery Disease Cases and Controls in the Case-Control Study

Characteristic	No. (%)	
	Patients with coronary artery disease (n = 4896)	Control participants (n = 5279)
Age, mean (SD), y	58.6 (7.2)	58.5 (7.2)
Male sex	3295 (67.3)	3533 (66.9)
Race and ethnicity		
European	4615 (94.3)	5118 (97.0)
African	45 (0.9)	42 (0.8)
East Asian	3 (0.1)	14 (0.3)
South Asian	151 (3.1)	46 (0.9)
Other^a^	82 (1.7)	59 (1.1)
Carriers of familial hypercholesterolemia variants	35 (0.7)	12 (0.2)
Lifestyle characteristic		
Healthy diet	1391 (28.4)	2491 (47.2)
Regular exercise	2217 (45.3)	2735 (51.8)
Not smoking	4013 (82.0)	4844 (91.8)
Absence of obesity	2934 (59.9)	4162 (78.8)
Lifestyle score, mean (SD)	2.16 (1.00)	2.70 (0.93)
Favorable	1796 (36.7)	3139 (59.5)
Intermediate	1764 (36.0)	1609 (30.5)
Unfavorable	1336 (27.3)	531 (10.1)

^a^ Included participants who answered “mixed,” “other ethnic group,” “do not know,” or “prefer not to answer.”

### Risk of CAD Among Carriers of an FH Variant and Noncarriers According to Lifestyle

**Figure 2.**
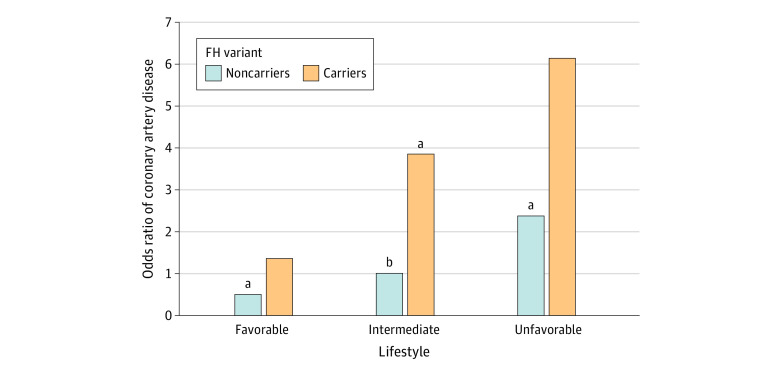
Risk of Coronary Artery Disease by Familial Hypercholesterolemia (FH) Variant Carrier Status and Adherence to Healthy Lifestyle in the Case-Control Study (N = 10 175) For each FH variant carrier status and lifestyle score group (coded as an indicator variable with 6 levels, 2 carrier status × 3 lifestyle groups), the odds ratio for disease was calculated in a logistic regression model with age, sex, and genetic ancestry as defined by the first 4 genetic principal components as covariates, with FH variant noncarriers with an intermediate lifestyle as the reference group. ^a^*P* < .05 in comparison with the reference group. ^b^Reference group.

The risk of CAD varied substantially among carriers and noncarriers according to healthy lifestyle characteristics. Compared with noncarriers with an intermediate lifestyle, carriers with a favorable lifestyle had an adjusted OR of 1.4 (95% CI, 0.5-3.5) (*P* = .53; Figure 2). By contrast, carriers with an unfavorable lifestyle had an adjusted OR of 6.1 (95% CI, 0.7-49.9) (*P* = .09). Within the limitations of statistical power, we did not observe a significant multiplicative interaction between FH carrier status and lifestyle score (OR, 1.2 [95% CI, 0.6-2.5]; *P* = .62 for interaction) or between FH carrier status and sex (OR, 1.2 [95% CI, 0.3-4.5]; *P* = .82 for interaction). We also did not observe a statistically significant additive interaction between FH carrier status and lifestyle score; the relative excess risk due to interaction was −0.18 (95% CI, −2.32 to 1.95).

We next sought to generalize the results from the case-control study to the independent cohort study of 39 920 participants. We identified a FH variant among 108 participants (0.3%) (Table 2; eTable 6 in the Supplement). Among these 108 carriers, the mean (SD) estimated untreated LDL cholesterol concentration was 200 (47) mg/dL compared with 145 (33) mg/dL in noncarriers (adjusted difference, 55 mg/dL [95% CI, 52-58 mg/dL]; *P* < .001). With respect to CAD, 22 of the 108 carriers (20.4%) developed CAD compared with 2832 of 39 812 noncarriers (7.1%), corresponding to an adjusted hazard ratio (HR) of 3.8 (95% CI, 2.5-5.8) (*P* < .001).

**Table 2.** Characteristics of FH Variant Carriers and Noncarriers in the Cohort Study

Characteristic	No. (%)	
	FH variant carriers (n = 108)	Noncarriers (n = 39 812)
Age at end of follow-up, mean (SD), y	66.5 (7.9)	66.4 (8.0)
Male sex	40 (37.0)	18 762 (47.1)
Hypertension	42 (38.9)	12 723 (32.0)
Diabetes	9 (8.3)	2668 (6.7)
Diagnosis of hypercholesterolemia	62 (57.4)	6100 (15.3)
Taking lipid-lowering medication	63 (58.3)	7586 (19.1)
LDL cholesterol, mean (SD), mg/dL		
Measured	163 (51)	137 (33)
Estimated untreated	200 (47)	145 (33)
Lifestyle characteristic		
Healthy diet	56 (51.9)	19 142 (48.1)
Regular exercise	55 (50.9)	20 472 (51.4)
Not smoking	97 (89.8)	36 361 (91.3)
Absence of obesity	76 (70.4)	30 557 (76.8)
Lifestyle score, mean (SD)	2.63 (1.12)	2.68 (0.96)
Favorable	66 (61.1)	23 331 (58.6)
Intermediate	22 (20.4)	11 830 (29.7)
Unfavorable	20 (18.5)	4651 (11.7)

Abbreviations: FH, familial hypercholesterolemia; LDL, low-density lipoprotein.

SI conversion factor: To convert LDL cholesterol to millimoles per liter, multiply by 0.0259.

As in the case-control study, each of the 4 healthy lifestyle characteristics was strongly associated with protection from CAD (healthy diet: HR, 0.59 [95% CI, 0.55-0.64]; regular exercise: HR, 0.77 [95% CI, 0.72-0.84]; not smoking: HR, 0.64 [95% CI, 0.60-0.68]; and absence of obesity: HR, 0.54 [95% CI, 0.50-0.58]) (eTable 5 in the Supplement), and we did not observe a significant interaction between FH carrier status and lifestyle score (HR, 0.8 [95% CI, 0.5-1.1]; *P* = .12 for interaction). When considered in aggregate lifestyle categories, 1272 of 23 397 participants (5.4%) with a favorable lifestyle developed CAD compared with 578 of 4671 participants (12.4%) with an unfavorable lifestyle, corresponding to an adjusted HR of 0.39 (95% CI, 0.36-0.43) (*P* < .001). This risk gradient according to lifestyle persisted even among those who carried a FH variant, for which a favorable lifestyle conferred 86% lower risk of CAD (adjusted HR, 0.14 [95% CI, 0.04-0.41]; *P* < .001).

### Age-Dependent Probability of CAD According to FH Variant and Lifestyle

**Figure 3.**
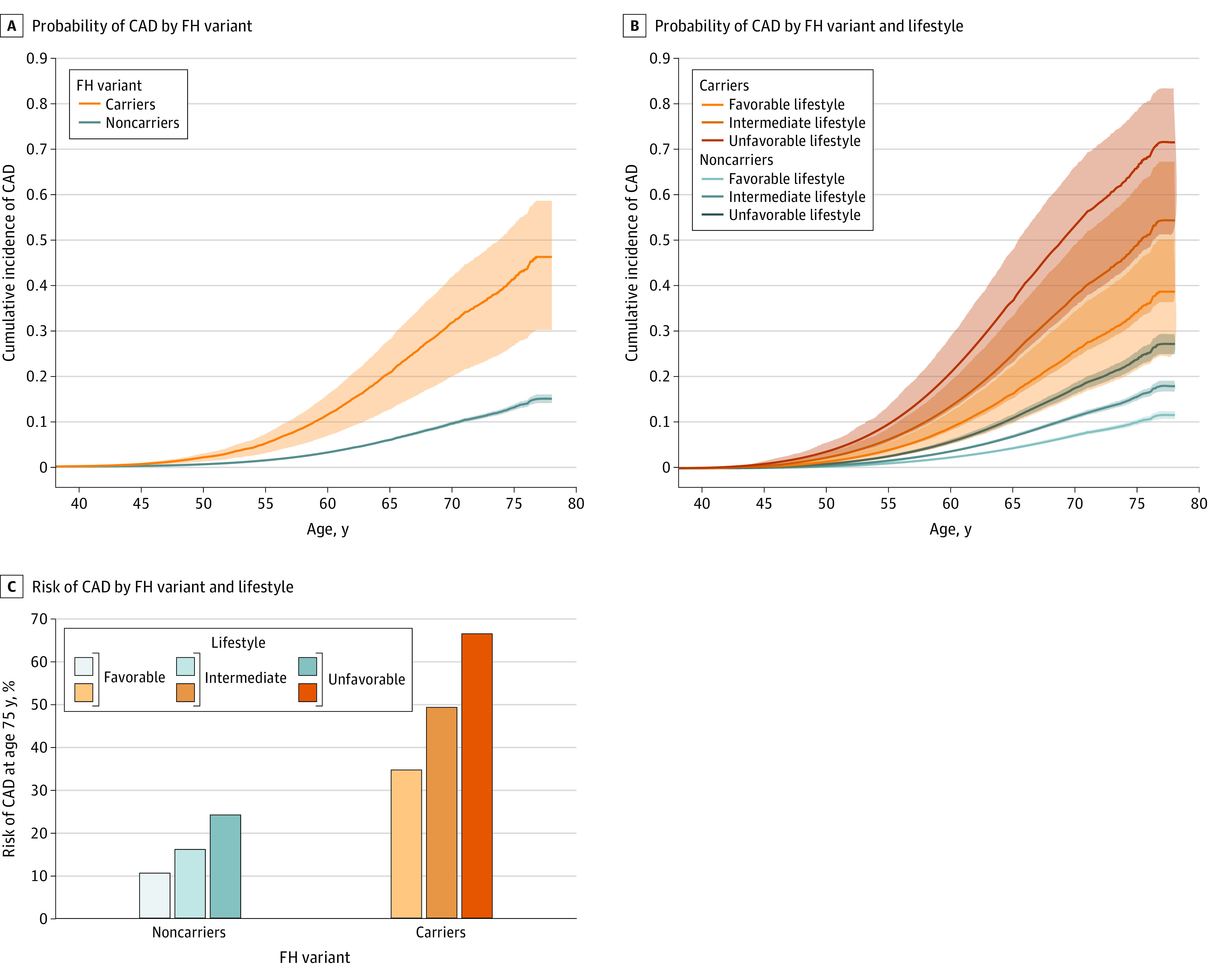
Probability of Coronary Artery Disease (CAD) by Familial Hypercholesterolemia (FH) Variant Carrier Status and Adherence to Healthy Lifestyle in the Cohort Study (N = 39 920) A, Age-dependent probability of CAD by FH variant carrier status. B, Age-dependent probability of CAD in FH carriers and noncarriers of different lifestyle scores. C, Estimated risk of CAD by the age of 75 years by FH variant carrier status and favorability of lifestyle. Age-dependent probability was quantified using a Cox proportional hazards regression model adjusted for sex and the first 4 genetic principal components, with the model standardized to the mean of each of the covariates. The shaded areas around the graph show the 95% CI.

Using a Cox proportional hazards regression model, we next estimated the age-dependent probability of manifesting CAD across strata of FH variants and lifestyle characteristics, noting a gradient of risk by the age of 75 years, ranging from 10.2% for those with no FH variant and a favorable lifestyle to 24.0% among those with no FH variant and an unfavorable lifestyle and from 34.5% among those with an FH variant and a favorable lifestyle to 66.2% for those with both an FH variant and an unfavorable lifestyle (Figure 3). The estimated absolute risk reduction for individuals with a favorable lifestyle compared with those with an unfavorable lifestyle was more than double for carriers (31.7%) compared with noncarriers (13.8%) (Figure 3C).

### Gaps in Recognition and Treatment of FH Variant Carriers

Even within the context of contemporary care and a national health system in the UK, our analysis highlighted important gaps in recognition and treatment of those who carry an FH variant. Among 82 carriers of an FH variant with no known CAD at time of enrollment and measured cholesterol levels, 62 had estimated untreated LDL cholesterol of at least 130 mg/dL. However, only 30 of these 62 individuals (48.4%) self-reported a history of hypercholesterolemia, and only 26 (41.9%) reported the use of lipid-lowering medications. Moreover, despite clinical recommendations that adherence to a healthy lifestyle is important among those with FH given their increased genetic risk of CAD, we observed that only 50.9% (55 of 108) reported regular exercise, 51.9% (56 of 108) followed a healthy diet pattern, 70.4% (76 of 108) maintained a body mass index in the nonobese range, and 56.5% (61 of 108) reported not smoking (Table 2).

## Discussion

In this analysis of genetic and lifestyle data of a case-control study (N = 10 175) and a cohort study (N = 39 930) derived from the UK Biobank, we found that carriers of FH variants had a mean 3-fold increased risk of CAD but that this risk varied significantly according to healthy lifestyle characteristics. This analysis is, to our knowledge, among the first to analyze the association of healthy lifestyle characteristics with risk of CAD in carriers of a FH variant compared with noncarriers within the context of contemporary care, confirming and extending similar observations within the general population or among those with high polygenic risk.^18^ We estimated that the probability of CAD among carriers of a FH variant ranged from 3.6% for those with a favorable lifestyle to 9.6% for those with an unfavorable lifestyle by the age of 55 years and ranged from 34.5% for those with a favorable lifestyle to 66.2% for those with an unfavorable lifestyle by the age of 75 years.

These results have at least 3 implications. First, carriers of pathogenic or likely pathogenic variants remain underdiagnosed and undertreated within current clinical care.^9,10,11^ Among those with both an FH variant and measured LDL cholesterol of at least 130 mg/dL, only 30 of 62 (48.4%) reported a history of hypercholesterolemia in an interview with a trained nurse at the time of enrollment. Moreover, at the time of study enrollment, the mean LDL cholesterol level among carriers of an FH variant was 167 mg/dL, with only 2 of 82 (2.4%) achieving the recommended target of less than 100 mg/dL.^34,35,36^ These results are consistent with the experience within the Geisinger Health System where, even after disclosure of an FH variant to carriers and their physicians, only 22% of carriers met LDL treatment goals.^37^ Identification of carriers of pathogenic variants based on clinical characteristics of LDL cholesterol levels alone remains challenging given the broad overlap with noncarriers, highlighting the potential value of the “genome-first” approach for systematic identification and disclosure now being piloted in several large studies.^38,39,40,41^

Second, we have added to a growing body of evidence regarding the strong genetic and nongenetic modifiers of the risk conferred by FH variants. We found that, despite a minimal association between adherence to a healthy lifestyle and LDL cholesterol concentrations, adherence to a healthy lifestyle may mitigate the risk of CAD. These findings reinforce previous studies that have similarly noted obesity and smoking as key lifestyle characteristics associated with cardiovascular disease among patients with FH.^42,43,44,45^ Beyond lifestyle, we and others have previously noted that measured LDL cholesterol concentrations, clinical risk factors such as diabetes or hypertension, peak exercise capacity, and polygenic background (with the risk conferred by the cumulative effect of many common DNA variants scattered across the genome) are each associated with CAD risk among those with FH variants.^3,22,46,47^ These results highlight the need to build on the seminal work by the SAFEHEART (Spanish Familial Hypercholesterolemia Cohort Study) registry investigators and others to develop integrative risk models for patients with FH variants to guide shared decision-making, ideally validated across care settings and diverse populations.^42^

Third, this work lends empirical support to lifestyle management recommendations for FH carriers. Current guidelines suggest that, beyond focusing on lowering of LDL cholesterol, avoidance of tobacco, a healthy diet pattern, and regular activity are key pillars of clinical management for those with FH variants.^36,48^ However, in our study, healthy lifestyle characteristics were similar among carriers and noncarriers of an FH variant. In our analysis, we observed prevalence ranging from 50% to 70% for each of 4 healthy lifestyle characteristics among those who carried an FH variant, with 40% having an intermediate or unfavorable lifestyle rating. The absence of a statistically significant interaction between carrier status and lifestyle score in this study suggests that lifestyle interventions are equally important in mitigating risk of CAD in carriers and noncarriers. However, the absolute risk reduction of CAD by healthy lifestyle among FH carriers was more than double that of noncarriers. From a clinical perspective, carriers of FH variants are at least at a 3-fold increased risk of CAD, and given that they remain underdiagnosed and undertreated, there is an opportunity for targeted intervention to promote adherence to healthy lifestyle measures. With the increasing availability of genomic information to inform risk, it is critical that communicating DNA-based risk estimates does not have to result in anxiety or fear.^19^ Shared decision-making informed by the potential of lifestyle change to attenuate risk may help alleviate any feelings of determinism.

### Limitations

This study has some limitations. The primary limitation was the observational study design. Our data, taken together with similar results in the population at large, provide evidence that a healthy lifestyle may mitigate inborn risk conferred by an FH variant. However, enabling change in patients’ lifestyle via targeted interventions has proven challenging both for carriers of FH variants and for other high-risk groups. Important examples include several randomized clinical trials that failed to meaningfully affect LDL cholesterol levels via a dietary intervention^49^ or improve cardiometabolic parameters significantly with multifaceted lifestyle interventions.^50,51^ Enhanced educational and motivational tools—ideally those that can be studied in a randomized fashion to quantify effect—remain an important public health need for those with FH.^10^ Additional limitations of this study were the definition of a healthy lifestyle and the lack of generalizability of the estimates to other populations. Although the definition of a healthy lifestyle follows guideline-based definitions for exercise and healthy diet, and the 4-point scoring system has been used repeatedly by our group and others,^18,25,26^ it is not validated prospectively, and there might be variations in the way a healthy lifestyle is measured in other studies. The UK Biobank also consists predominantly of individuals of European ancestry and is on average healthier than the general population, due to a “healthy volunteer” bias.^52^ As such, absolute disease estimates are likely to vary and would need to be calibrated to other more diverse populations.

## Conclusions

Using genetic and lifestyle data from a case-control study and a cohort study derived from the UK Biobank, this study suggests that carriers of FH variants have at least a 3-fold increased risk of CAD but that a favorable lifestyle is associated with a lower risk of CAD in both FH carriers and noncarriers despite minimal association with LDL cholesterol levels. Because carriers of FH variants remain underdiagnosed and undertreated in contemporary practice, identifying and targeting those carriers with interventions to enhance adherence to a healthy lifestyle may provide an opportunity to reduce their risk of CAD.

## Notes

Supplement. **eTable 1.** Characteristics of the Case-Control Study Participants Compared With Excluded Participants due to Incomplete Lifestyle Data **eTable 2.** Characteristics of the Cohort Study Participants Compared With Excluded Participants due to Incomplete Lifestyle Data **eTable 3.** Adjustment of LDL Cholesterol Levels in the UK Biobank Based on Self-Report of LDL Cholesterol–Lowering Medications **eTable 4.** List of Pathogenic or Likely Pathogenic Familial Hypercholesterolemia Variants in the Case-Control Study (N=10,715) **eTable 5.** Association of Healthy Lifestyle Factors With Incident CAD in the UK Biobank (N=39,920) **eTable 6.** List of Pathogenic or Likely Pathogenic Classifications of Familial Hypercholesterolemia Variants in the Cohort Study (N=39,920) **eFigure 1.** Study Diagram **eFigure 2.** Distribution of Healthy Lifestyle Score Values in Cases and in Controls (N=10,715)
eReferences.
