# Supplementary material for: Association of the Interaction Between Familial Hypercholesterolemia Variants and Adherence to a Healthy Lifestyle With Risk of Coronary Artery Disease
Source: JAMA Netw Open. 2022 Mar 16;5(3):e222687. doi: 10.1001/jamanetworkopen.2022.2687 (PMC8928007; doi:10.1001/jamanetworkopen.2022.2687)
Supplement: Supplement. — eTable 1. Characteristics of the Case-Control Study Participants Compared With Excluded Participants due to Incomplete Lifestyle Data eTable 2. Characteristics of the Cohort Study Participants Compared With Excluded Participants due to Incomplete Lifestyle Data eTable 3. Adjustment of LDL Cholesterol Levels in the UK Biobank Based on Self-Report of LDL Cholesterol–Lowering Medications eTable 4. List of Pathogenic or Likely Pathogenic Familial Hypercholesterolemia Variants in the Case-Control Study (N=10,715) eTable 5. Association of Healthy Lifestyle Factors With Incident CAD in the UK Biobank (N=39,920) eTable 6. List of Pathogenic or Likely Pathogenic Classifications of Familial Hypercholesterolemia Variants in the Cohort Study (N=39,920) eFigure 1. Study Diagram eFigure 2. Distribution of Healthy Lifestyle Score Values in Cases and in Controls (N=10,715) eReferences. [file jamanetwopen-e222687-s001.pdf]

Supplementary Online Content

Fahed AC, Wang M, Patel AP, et al. Association of the interaction between familial hypercholesterolemia variants and adherence to a healthy lifestyle with risk of coronary artery disease. *JAMA Netw Open.* 2022;5(3):e222687.

doi:10.1001/jamanetworkopen.2022.2687

eTable 1. Characteristics of the Case-Control Study Participants Compared With Excluded Participants due to Incomplete Lifestyle Data
eTable 2. Characteristics of the Cohort Study Participants Compared With Excluded Participants due to Incomplete Lifestyle Data
eTable 3. Adjustment of LDL Cholesterol Levels in the UK Biobank Based on Self-Report of LDL Cholesterol–Lowering Medications
eTable 4. List of Pathogenic or Likely Pathogenic Familial Hypercholesterolemia Variants in the Case-Control Study (N=10,715)
eTable 5. Association of Healthy Lifestyle Factors With Incident CAD in the UK Biobank (N=39,920)
eTable 6. List of Pathogenic or Likely Pathogenic Classifications of Familial Hypercholesterolemia Variants in the Cohort Study (N=39,920)
eFigure 1. Study Diagram
eFigure 2. Distribution of Healthy Lifestyle Score Values in Cases and in Controls (N=10,715)
eReferences.

This supplementary material has been provided by the authors to give readers additional information about their work.

© 2022 Fahed AC et al. *JAMA Network Open.*

eTable 1. Characteristics of the Case-Control Study Participants Compared With Excluded Participants due to Incomplete Lifestyle Data

	Study participants (N=10,175)	Excluded participants due to incomplete lifestyle data (N=2,677)
Age, mean (SD), years	58.6 (7.2)	59.5 (7.1)
Male sex, n (%)	6,828 (67.1)	1,542 (57.6)
Coronary artery disease, n (%)	4,896 (48.1)	1,536 (57.4)
Carriers of Familial Hypercholesterolemia variants, n (%)	47 (0.5)	9 (0.3)

© 2022 Fahed AC et al. *JAMA Network Open.*

eTable 2. Characteristics of the Cohort Study Participants Compared With Excluded Participants due to Incomplete Lifestyle Data

	Study participants (N=39,920)	Excluded participants due to incomplete lifestyle data (N=8,892)
Age at the end of follow-up mean (SD), years	66.4 (8.0)	67.8 (7.8)
Male sex, n (%)	18,802 (47.1)	3413 (38.4)
Coronary artery disease, n (%)	2,854 (7.1)	793 (8.9)
Carriers of Familial Hypercholesterolemia variants, n (%)	108 (0.3)	22 (0.2)

© 2022 Fahed AC et al. *JAMA Network Open.*

eTable 3. Adjustment of LDL Cholesterol Levels in the UK Biobank Based on Self-Report of LDL Cholesterol–Lowering Medications [a]

Medication	LDL Cholesterol Adjustment	Reference(s)
Statin	-30%	[1]
Ezetimibe	-20%	[2–4]
Bile Acid Sequestrant	-15%	[5]
Fibrate	-10%	[6]
Niacin	-10%	[6]
Not specified*	-30%	[1]

[a] We curated self-report of LDL cholesterol-lowering medication at enrollment in two ways. First, participants were asked "Do you regularly take cholesterol lowering medication?" Second, participants were asked to provide a list of all medications they are currently taking, which we coded and converted to the categories listed in this table. In participants who self-reported taking LDL cholesterol-lowering medications at enrollment, measured LDL cholesterol was adjusted depending on the type of lipid-lowering medication intake based on prior reports of effect size for each medication type from the literature. For example, in the case of statin intake, LDL cholesterol was divided by 0.7 by in the case of ezetimibe intake, LDL cholesterol was divided by 0.8. When the lipid-lowering medication was not specified, it was assumed a statin. We conducted a sensitivity analysis to test the robustness of our estimation of untreated LDL cholesterol in a subset of 1,791 UK Biobank participants who had untreated LDL cholesterol measured at enrollment in the UK Biobank between 2006 and 2010, and a repeat measurement on treatment on a follow-up assessment after 2012. The mean LDL cholesterol levels were as follows: untreated baseline 156±33 mg/dL, treated follow-up 118±33 mg/dL, and estimated untreated based on follow-up measurement 150±36 mg/dL.

© 2022 Fahed AC et al. *JAMA Network Open.*

eTable 4. List of Pathogenic or Likely Pathogenic Familial Hypercholesterolemia Variants in the Case-Control Study (N=10,715) [a]

Variant	Gene (Variant Type)	Amino acid or cDNA change	Number of carriers
2:21229161:G>A	*APOB* Missense	p.Arg3527Trp	1
2:21229160:C>T	*APOB* Missense	p.Arg3527Gln	11
19:11213390:C>T	*LDLR* Missense	p.Arg81Cys	1
19:11216084:G>A	*LDLR* Missense	p.Asp168Asn	1
19:11216133:G>A	*LDLR* Missense	p.Cys184Tyr	1
19:11216244:A>G	*LDLR* Missense	p.Asp221Gly	1
19:11216262:AC>*	*LDLR* Frameshift	p.Asp227Glyfs*12	1
19:11216264:G>T	*LDLR* Premature stop	p.Glu228*	2
19:11217264:G>A	*LDLR* Missense	p.Glu240Lys	1
19:11221414:G>A	*LDLR* Missense	p.Gly343Ser	2
19:11224013:C>T	*LDLR* Missense	p.Arg416Trp	1
19:11213408:T>G	*LDLR* Missense	p.Trp87Gly	1
19:11224266:G>T	*LDLR* Missense	p.Asp472Tyr	1
19:11230819:C>T	*LDLR* Missense	p.Arg633Cys	3
19:11231112:C>T	*LDLR* Missense	p.Pro685Leu	2
19:11215919:G>A	*LDLR* Missense	p.Glu113Lys	4
19:11213452:G>*	*LDLR* Premature stop	p.Glu101Aspfs*105	1
19:11224296:G>A	*LDLR* Missense	p.Asp482Asn	5
19:11216146:C>G	*LDLR* Premature stop	p.Tyr188*	1
19:11223944:G>A	*LDLR* Splice site	c.1187-10G>A	1
19:11226820:G>A	*LDLR* Missense	p.Gly546Asp	1
19:11227549:C>T	*LDLR* Missense	p.Arg574Cys	1
19:11230767:G>A	*LDLR* Splice site	c.1846-1G>A	1
1:55523127:G>T	*PCSK9* Missense	p.Asp374Tyr	1

[a] Evidence of pathogenicity of each variant is reported in a previous manuscript.[7]

© 2022 Fahed AC et al. *JAMA Network Open.*

eTable 5. Association of Healthy Lifestyle Factors With Incident CAD in the UK Biobank (N=39,920)

Healthy Lifestyle Characteristic	N (%) in 2854 who developed CAD	N (%) in 37,066 free of CAD	HR (95% CI) for CAD	P-value
Healthy diet	1,013 (35%)	18,185 (49%)	0.59 (0.55-0.64)	$<2\ x10^{-16}$
Regular exercise	1,341 (47%)	19,186 (52%)	0.77 (0.72-0.84)	$<2x10^{-16}$
Not smoking	2352 (89%)	33,926 (99%)	0.64 (0.60-0.68)	$9x10^{-14}$
Absence of obesity	1,836 (64%)	28,797 (78%)	0.54 (0.50-0.58)	$< 2x10^{-16}$

© 2022 Fahed AC et al. *JAMA Network Open.*

eTable 6. List of Pathogenic or Likely Pathogenic Classifications of Familial Hypercholesterolemia Variants in the Cohort Study (N=39,920) [a]

Variant	Gene (Variant Type)	Amino acid or cDNA change	Number of carriers
1:55505604:G>A	PCSK9 Missense	p.Glu32Lys	1
1:55523127:G>T	PCSK9 Missense	p.Asp374Tyr	1
1:55524303:C>T	PCSK9 Missense	p.Arg496Trp	5
2:21229160:C>T	APOB Missense	p.Arg3527Gln	25
2:21229161:G>A	APOB Missense	p.Arg3527Trp	2
19:11210949:A>*	LDLR Frameshift	p.Ile40SerfsX166	1
19:11213360:G>*	LDLR Frameshift	p.Asp72ThrfsX134	1
19:11213390:C>T	LDLR Missense	p.Arg81Cys	1
19:11213408:T>G	LDLR Missense	p.Trp87Gly	2
19:11213415:G>A	LDLR Missense	p.Cys89Tyr	2
19:11213450:G>A	LDLR Missense	p.Glu101Lys	6
19:11213462:CG>*	LDLR splice site	c.313_313+1delCG	1
19:11213463:G>C	LDLR splice site	c.313+1G>C	1
19:11213463:G>A	LDLR splice site	c.313+1G>A	1
19:11215919:G>A	LDLR Missense	p.Glu113Lys	4
19:11216083:C>A	LDLR Nonsense	p.Cys167X	1
19:11216084:G>A	LDLR Missense	p.Asp168Asn	3
19:11216233:TGG>*	LDLR Deletion	p.Gly219del	1
19:11216244:A>G	LDLR Missense	p.Asp221Gly	3
19:11216262:AC>*	LDLR Frameshift	p.Asp227GlyfsX12	1
19:11216263:C>G	LDLR Missense	p.Asp227Glu	1
19:11216264:G>T	LDLR Nonsense	p.Glu228X	1
19:11217264:G>A	LDLR Missense	p.Glu240Lys	2
19:11217344:T>A	LDLR Missense	p.Asp266Glu	1
19:11218112:G>A	LDLR Missense	p.Glu288Lys	1
19:11218162:C>G	LDLR Missense	p.Asp304Glu	1
19:11221390:G>A	LDLR Missense	p.Gly335Ser	2
19:11221435:C>T	LDLR Nonsense	p.Arg350X	2
19:11223983:C>T	LDLR Missense	p.Arg406Trp	1
19:11223989:G>A	LDLR Missense	p.Glu408Lys	1
19:11224052:G>A	LDLR Missense	p.Val429Met	1
19:11224266:G>T	LDLR Missense	p.Asp472Tyr	3
19:11224296:G>A	LDLR Missense	p.Asp482Asn	3
19:11224326:G>A	LDLR Missense	p.Asp492Asn	1
19:11224419:G>A	LDLR Missense	p.Val523Met	1

© 2022 Fahed AC et al. *JAMA Network Open.*

19:11224443:G>A	LDLR splice site	c.1586+5G>A	3
19:11226816:G>*	LDLR Frameshift	p.Gly546AlafsX2	1
19:11226817:G>A	LDLR Missense	p.Gly545Glu	4
19:11226820:G>T	LDLR Missense	p.Gly546Val	1
19:11227549:C>T	LDLR Missense	p.Arg574Cys	3
19:11227612:C>T	LDLR Missense	p.Arg595Trp	1
19:11230819:C>T	LDLR Missense	p.Arg633Cys	2
19:11230820:G>A	LDLR Missense	p.Arg633His	1
19:11231095:T>A	LDLR Nonsense	p.Tyr679X	1
19:11231112:C>T	LDLR Missense	p.Pro685Leu	6

[a] Evidence of pathogenicity of each variant is reported in a previous manuscript.[7]

© 2022 Fahed AC et al. *JAMA Network Open.*

eFigure 1. Study Diagram

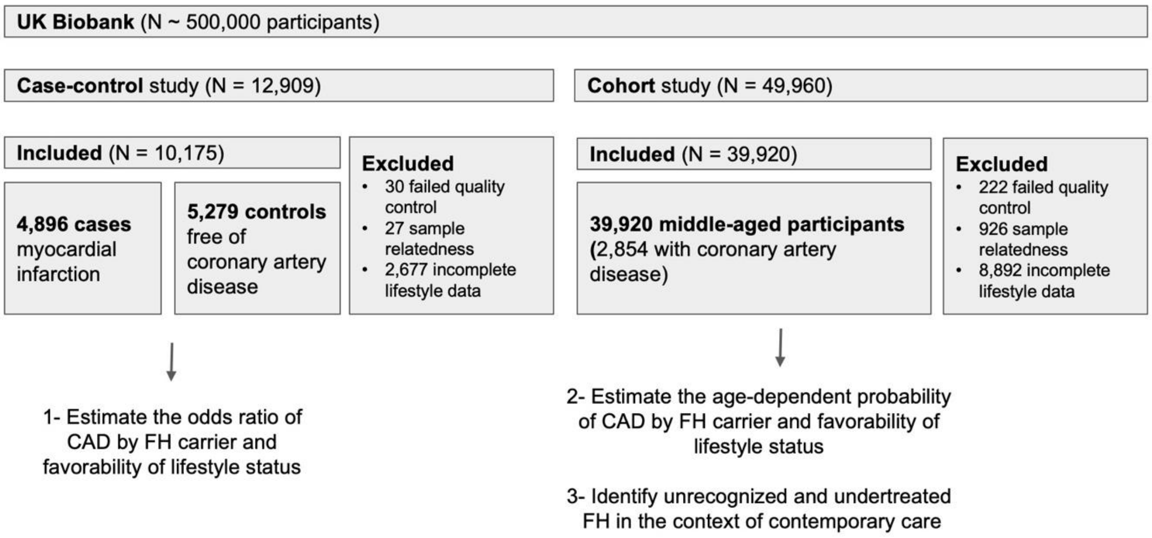

© 2022 Fahed AC et al. *JAMA Network Open.*

eFigure 2. Distribution of Healthy Lifestyle Score Values in Cases and in Controls (N=10,715)

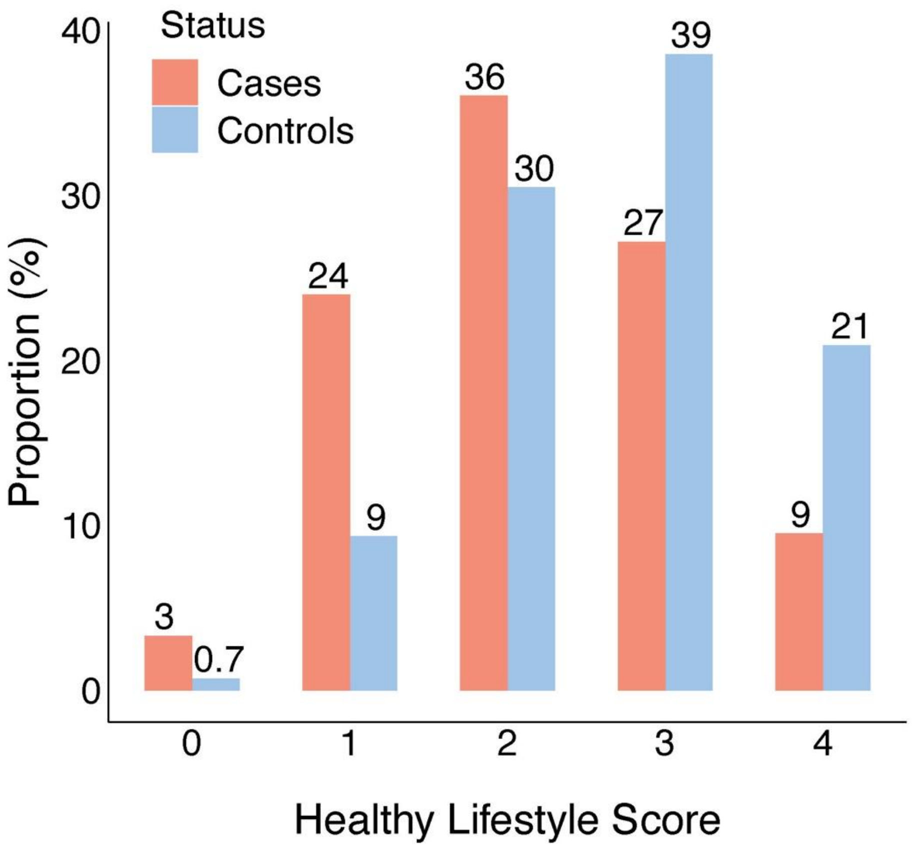

© 2022 Fahed AC et al. *JAMA Network Open.*

eReferences.

1. Cholesterol Treatment Trialists' (CTT) Collaboration. Efficacy and safety of more intensive lowering of LDL cholesterol: a meta-analysis of data from 170 000 participants in 26 randomised trials. *The Lancet*. 2010;376(9753):1670-1681. doi:10.1016/s0140-6736(10)61350-5

2. Sudhop T, Lütjohann D, Kodal A, et al. Inhibition of Intestinal Cholesterol Absorption by Ezetimibe in Humans. *Circulation*. 2002;106(15):1943-1948. doi:10.1161/01.Cir.0000034044.95911.Dc

3. Cannon CP, Blazing MA, Giugliano RP, et al. Ezetimibe Added to Statin Therapy after Acute Coronary Syndromes. *N Engl J Med*. 2015;372(25):2387-2397. doi:10.1056/NEJMoa1410489

4. Zhao Z, Du S, Shen S, et al. Comparative efficacy and safety of lipid-lowering agents in patients with hypercholesterolemia: A frequentist network meta-analysis. *Med Baltim*. 2019;98(6):e14400. doi:10.1097/MD.0000000000014400

5. Lloyd-Jones DM, Morris PB, Ballantyne CM, et al. 2017 Focused Update of the 2016 ACC Expert Consensus Decision Pathway on the Role of Non-Statin Therapies for LDL-Cholesterol Lowering in the Management of Atherosclerotic Cardiovascular Disease Risk: A Report of the American College of Cardiology Task Force on Expert Consensus Decision Pathways. *J Am Coll Cardiol*. 2017;70(14):1785-1822. doi:10.1016/j.jacc.2017.07.745

6. Birjmohun RS, Hutten BA, Kastelein JJ, Stroes ES. Efficacy and safety of high-density lipoprotein cholesterol-increasing compounds: a meta-analysis of randomized controlled trials. *J Am Coll Cardiol*. 2005;45(2):185-197. doi:10.1016/j.jacc.2004.10.031

7. Fahed AC, Wang M, Homburger JR, et al. Polygenic background modifies penetrance of monogenic variants for tier 1 genomic conditions. *Nat Commun*. 2020;11(1):3635. doi:10.1038/s41467-020-17374-3

© 2022 Fahed AC et al. *JAMA Network Open.*
